# Serotypes, sequence types, and erythromycin resistance of maternal colonizing group B *Streptococcus*: implications for neonatal early-onset disease prevention

**DOI:** 10.3389/fped.2026.1952830

**Published:** 2026-09-03

**Authors:** Xia Sun, Hua Liu

**Affiliations:** 1 School of Clinical Medicine, Shandong Second Medical University, Weifang, Shandong, China; 2 Department of Obstetrics, Gaomi Maternity and Child Health Hospital, Gaomi, Shandong, China; 3 Department of Gynecology, Affiliated Hospital of Shandong Second Medical University, Weifang, Shandong, China

**Keywords:** erythromycin resistance, group B *Streptococcus*, inducible clindamycin resistance, intrapartum antibiotic prophylaxis, multilocus sequence typing

## Abstract

Intrapartum antibiotic prophylaxis for maternal group B *Streptococcus* (GBS) colonization depends on the assumption that a woman identified as colonized receives an antibiotic active against her own isolate, an assumption that routine susceptibility reporting may overestimate when inducible clindamycin resistance is present. This study characterized the capsular serotypes, sequence types, macrolide-lincosamide resistance phenotypes and the intrapartum antibiotic actually administered for maternal colonizing GBS isolates at a single tertiary maternity centre. Banked isolates from antenatal screening were revived and analysed by capsular multiplex PCR, multilocus sequence typing, broth microdilution, D-test and resistance gene PCR, and each isolate was linked to the intrapartum regimen recorded in the corresponding delivery record. Among 120 isolates, serotype III predominated at 35.8%, and the six serotypes included in a hexavalent capsular vaccine accounted for 94.9% of typeable isolates, representing a serotype-based ceiling rather than expected vaccine effectiveness. Thirteen sequence types were resolved, and CC17/ST17 accounted for 11.7% of the collection. All isolates were susceptible to penicillin, ampicillin and vancomycin, whereas erythromycin non-susceptibility reached 63.3%, comprising constitutive (40.0%), inducible (8.3%) and M (15.0%) phenotypes, with a further 3.3% showing the L phenotype. Routine testing classified 56.7% of isolates as clindamycin-susceptible, but effective *in-vitro* clindamycin availability after reclassification of D-test-positive isolates was 48.3%, an absolute reduction of 8.3 percentage points. The inducible phenotype was more frequent in CC17/ST17 than in other lineages (28.6% versus 5.7%), although this subgroup contained only 14 isolates and should be interpreted as exploratory. Among the women who had actually received clindamycin during labour, half were colonized by isolates against which clindamycin lacked *in-vitro* activity. These findings support routine D-testing of erythromycin non-susceptible maternal GBS isolates and reporting that states whether clindamycin is suitable for intrapartum prophylaxis.

## 1 Introduction

*Streptococcus agalactiae*, also known as group B *Streptococcus* (GBS), is the foremost bacterial cause of early-onset disease (EOD) in neonates, usually developing into sepsis or pneumonia during the first 7 days of their lives (1). The primary source of GBS infection is maternal recto-vaginal carriage, which affects about 8% of pregnant women in China and about 17% globally, and after vertical transmission of the infection, a small but consistent percentage of the babies develop invasive disease (2, 3). Once invasive infection occurs, there is an expected case fatality rate of around 5% in high-resource countries, while approximately one in five survivors of invasive GBS infection is subsequently diagnosed with a neurodevelopmental disorder, the risk being highest after meningitis (4, 5). Thus, prevention relies on identifying the presence of the carrier women before birth and preventing the infection during labor. The approach recommended by the American College of Obstetricians and Gynecologists includes universal testing for GBS at 36 0/7 to 37 6/7 weeks of gestation and intrapartum antibiotic prophylaxis (IAP) for those who test positive, an approach linked with a decline in EOD prevalence higher than 80% compared to the era before prophylaxis (6). However, its continued success relies on two linked premises that are rarely examined together: that screening identifies the strain relevant to neonatal exposure, and that the administered intrapartum antibiotic is active against that isolate.

The risk to infants carried by colonizing GBS is not evenly distributed across its population structure. Serotype III is over-represented in invasive neonatal strains, and the hypervirulent clonal complex 17 (CC17), which is typically serotype III, accounted for 66% of neonatal invasive disease cases in a large national series (7). The enhanced virulence is partially due to the cell-surface adhesin HvgA, while integrative and conjugative elements associated with serotype III/ST17 strains endow them with multidrug resistance (7, 8). Routine screening reports only the presence of the species and cannot resolve this structure. The capsule composition also has some importance because there are vaccine candidates for serotypes Ia, Ib, II, III, IV, and V that have reached advanced clinical trials (9), and the regional prevalence of capsule types sets an upper bound on the proportion of disease such vaccines could prevent (3). In either case, typing results from colonizing isolates are essential.

Both penicillin and ampicillin are still preferred as treatment drugs for GBS, with this pathogen remaining essentially uniformly susceptible to β-lactams, although rare isolates with decreased susceptibility due to alterations in the *pbp2x* gene have been found through population studies (10). For women at high risk of anaphylaxis to penicillin, clindamycin is an alternative when susceptibility testing shows that the bacteria are susceptible to this agent (6). This condition is increasingly difficult to satisfy, because global resistance to erythromycin and clindamycin has risen steadily in recent years (11), and pooled erythromycin resistance among Chinese maternal isolates reaches 65.4% (2). Most of the resistance is due to *erm* genes coding for ribosomal methylases that are either constitutively (cMLSB) or inducibly (iMLSB) expressed (12). The inducible phenotype is particularly important for prophylaxis, because such isolates appear clindamycin-susceptible on broth microdilution and disc diffusion testing and are revealed only when erythromycin and clindamycin discs are placed close to each other, where the characteristic blunting of the clindamycin inhibition zone occurs (12, 13). Exposed to clindamycin, inducible isolates become constitutive, and treatment failure due to this form of resistance has been described repeatedly in Gram-positive pathogens (14). Without the D-test, isolates able to express resistance are reported as susceptible, and the percentage of women for whom clindamycin is a viable preventative measure is correspondingly overestimated.

These elements have not previously been reported together for this population: the serotype and sequence type composition of maternal colonizing isolates, the distribution of macrolide-lincosamide resistance phenotypes defined by D-test, and the linkage of resistance genotype to the antibiotic each colonized woman actually received during delivery. This study addresses all three in a single collection of maternal colonizing GBS isolates. In connecting each isolate to the treatment recorded in its respective delivery note, it also quantifies how often clindamycin prophylaxis already delivered at this institution was microbiologically ineffective against the woman's own isolate, a microbiological measure that is distinct from, and does not by itself establish, clinical failure in the neonate.

## 2 Materials and methods

### 2.1 Study design, setting and isolates

This study is a retrospective cohort study conducted within a single tertiary maternity centre. The study protocol was approved by the Ethics Committee of Gaomi Maternity and Child Health Hospital (approval number GM20231101). Informed consent was not required as the research employed retrospective use of banked isolates and clinical records. The study follows the guidelines of the Declaration of Helsinki and STROBE Statement guidelines for reporting of observational studies.

All the GBS isolates consecutively stored from antenatal screening procedures performed during January 2023 and December 2025 were sourced from the strain library maintained by the laboratory department of the study centre. Where there were several isolates stored from one patient during pregnancy, the earliest isolate alone was kept, and those isolates which did not have any connection to available delivery records were discarded. Isolates were collected using the standard GBS testing performed on pregnant patients at the hospital. A single flocked swab (Copan Diagnostics, Brescia, Italy) was inserted into the lower vagina and then into the anorectum between 36 0/7 and 37 6/7 weeks of gestation without the aid of a speculum. Swabs were then placed in the Amies transport medium and delivered to the clinical microbiology laboratory within 4 h. Each specimen was inoculated in 5 mL of LIM selective broth medium containing Todd Hewitt broth with 10 µg/mL colistin and 15 µg/mL nalidixic acid (Oxoid, Basingstoke, UK) and incubated for 18–24 h in 35 °C–37 °C and aerobic conditions. After growth in broth medium, the cultures were sub-cultured in Columbia Agar with 5% defibrinated sheep blood (bioMérieux, Marcy-l'Étoile, France) and were incubated again for 18–24 h in 35 °C–37 °C and aerobic conditions. Colonies with morphology and haemolysis consistent with GBS were subcultured and identified by the CAMP reaction, group B latex agglutination (Slidex Strepto Plus B, bioMérieux) and matrix-assisted laser desorption/ionization time-of-flight mass spectrometry on a MALDI Biotyper (Bruker Daltonics, Bremen, Germany), with in-house *cfb* gene PCR performed on isolates giving discordant or borderline results.

Isolates had been stored at −80 °C in cryogenic vials with glycerol as cryoprotectant (Microbank, Pro-Lab Diagnostics, Round Rock, TX, USA). The isolates were subcultured twice on Columbia agar plates enriched with 5% sheep blood after being revived, and the identification of species was confirmed using MALDI-TOF mass spectrometry before further testing. Isolates which did not grow after two subcultures or failed to give an identification of *Streptococcus agalactiae* after confirmation were not used in further analysis. Susceptibility testing and molecular assays were performed on the revived isolates.

### 2.2 Serotyping and multilocus sequence typing

Capsular typing was carried out using the dual multiplex PCR scheme proposed by Poyart et al. (15), which discriminates capsular types Ia, Ib and II–IX. Genomic DNA was extracted using the QIAamp DNA Mini Kit (Qiagen, Hilden, Germany), and amplification was performed with HotStarTaq Plus Master Mix (Qiagen) on a T100 thermal cycler (Bio-Rad Laboratories, Hercules, CA, USA). Gel electrophoresis using 2% agarose gels followed by GelRed staining (Biotium, Fremont, CA, USA) was used for product detection. Latex agglutination with the use of the ImmuLex Strep-B kit (SSI Diagnostica, Hillerød, Denmark) was used as the reference technique. Discrepancies between the results obtained by both techniques were resolved based on molecular typing (16). Non-typeable isolates were classified as NT.

Multilocus sequence typing was performed according to the scheme of Jones et al. (17), amplifying and sequencing the seven housekeeping genes *adhP*, *pheS*, *atr*, *glnA*, *sdhA*, *glcK* and *tkt*. Amplicons were purified using the QIAquick PCR Purification Kit (Qiagen) and sequenced on an ABI 3730xl DNA Analyzer (Applied Biosystems, Foster City, CA, USA). Allele numbers and sequence types were assigned by querying the PubMLST *Streptococcus agalactiae* database (https://pubmlst.org/organisms/streptococcus-agalactiae). The definition of clonal complexes was conducted based on the goeBURST algorithm using PHYLOViZ 2.0 (18) such that a clonal complex is composed of strains sharing six out of seven loci with the group founder.

### 2.3 Antimicrobial susceptibility testing and resistance genes

#### 2.3.1 MIC determination

Minimum inhibitory concentrations were determined using broth microdilution in cation-supplemented Mueller–Hinton broth containing 3% lysed horse blood using custom made Sensititre STP6F plates (Thermo Fisher Scientific, Waltham, MA, USA). The test was performed in accordance with the manufacturer's guidelines as well as the CLSI M100 supplement, 34th edition, 2024 (19). The panel covered penicillin, ampicillin, erythromycin, clindamycin, vancomycin, levofloxacin and tetracycline. Categorical interpretations were based on the *Streptococcus* spp. β-haemolytic group breakpoints of CLSI M100. *Streptococcus pneumoniae* ATCC 49619 was tested in every batch as the quality control organism, and only batches with control values within the acceptable range were retained.

#### 2.3.2 D-test and classification of macrolide-lincosamide resistance phenotypes

Inducible clindamycin resistance was assessed by the D-test as described in the CLSI M100 supplement (19). A 0.5 McFarland suspension of each isolate was inoculated onto Mueller–Hinton agar containing 5% sheep blood (Oxoid), and a 15 µg erythromycin disc (Oxoid) and a 2 µg clindamycin disc (Oxoid) were placed on the agar surface with their edges 12 mm apart. The plates were grown for 20–24 h at 35 °C in 5% CO_2_. This test was performed for each isolate which showed resistance or intermediate sensitivity to erythromycin. Each of the two readers interpreted the results without knowing what each other had recorded. In case of disagreement, a third reader decided on the result. The phenotypes were identified based on the criteria from Table 1.

**Table 1:** Macrolide-lincosamide resistance phenotype assignment.

Phenotype	Interpretation
cMLSB	Erythromycin-resistant and clindamycin-resistant, no D-shaped blunting
iMLSB	Erythromycin-resistant and clindamycin-susceptible, with flattening of the clindamycin inhibition zone facing the erythromycin disc
M	Erythromycin-resistant and clindamycin-susceptible, without blunting
L	Erythromycin-susceptible and clindamycin-resistant

#### 2.3.3 Detection of resistance genes

Genomic DNA extracted for MLST was also used to screen for the erythromycin ribosomal methylase genes *ermB* and *ermA* (including the *ermTR* subclass), the macrolide efflux determinant *mefA/E*, and the lincosamide resistance genes *lnuB* and *lsaC* by conventional PCR. Primer sequences, annealing temperatures and expected amplicon sizes followed Sutcliffe et al. (20) for the *erm* and *mef* targets and Malbruny et al. (21) for *lsaC*. The amplification was done using HotStarTaq Plus Master Mix in T100 Thermal Cycler. The PCR amplicons of the expected size were purified using the QIAquick PCR Purification Kit and sequenced using ABI 3730xl DNA Analyzer with the help of comparison with their respective GenBank sequences. The isolates where there is no correlation between their resistance genotypes and phenotypes were noted separately.

### 2.4 Clinical data and neonatal outcomes

The maternal factors identified from the delivery electronic chart record were age, gravida status, parity, gestational age at time of screening, gestational age at time of delivery, mode of delivery, rupture-to-delivery interval, prolonged rupture of membranes (≥18 h), intrapartum fever, clinical chorioamnionitis, preterm delivery, history and severity of penicillin allergy, the specific intrapartum antibiotic used (penicillin, ampicillin, cefazolin, clindamycin, or vancomycin), and the interval from the first antibiotic dose to delivery, recorded in hours and analysed both as a continuous variable and dichotomised at 4 h in accordance with (6). The neonatal factors were obtained from the newborn electronic chart record and included neonatal intensive care unit admission, neonatal sepsis evaluation, positive culture results from blood and cerebrospinal fluid cultures, and diagnosis of early-onset disease in the first 7 days of life. Virulence determinants such as *hvgA* were not genotyped, and CC17 membership was defined by MLST alone. These data were collected independently by two investigators using the case report form and any inconsistencies were sorted out through use of the primary record. The number of missing values was calculated for each variable. The records having missing values were kept for descriptive statistics purposes, but they were not included in the particular analysis of each individual variable without imputation.

### 2.5 Endpoints and derived indicators

The main measure was the percentage of strains that showed erythromycin non-susceptibility and, among those strains, the distribution across the four phenotypes of macrolide-lincosamide resistance. The potential coverage of the hexavalent vaccine was obtained as the ratio between the number of strains belonging to serotypes Ia, Ib, II, III, IV, or V and the total number of typeable strains. The percentage of the EOD-associated CC17/ST17 lineage was also calculated relative to all analyzed and typeable strains.

Effective clindamycin availability for intrapartum prophylaxis was defined microbiologically as one minus the sum of cMLSB, iMLSB, and L-phenotype isolates divided by the relevant denominator; M-phenotype isolates, which are erythromycin-resistant but retain clindamycin activity, were counted as available. This classification followed the guideline requirement that clindamycin be used for intrapartum prophylaxis only when the colonizing isolate is susceptible (6). This indicator was calculated for three denominators used in the results: all analysed isolates, penicillin-allergic women, and high-risk-allergic women. The estimates for the smaller denominators are descriptive.

High-risk-lineage clindamycin unavailability was calculated as the proportion of CC17/ST17 isolates carrying the cMLSB, iMLSB, or L phenotype. Microbiologically ineffective clindamycin prophylaxis was defined, among women who received clindamycin, as colonisation by an isolate classified as iMLSB, cMLSB, or L; it denotes absence of *in-vitro* activity of the administered agent against the woman's own isolate and is distinct from clinical failure in the neonate, which was assessed separately as culture-confirmed early-onset disease.

### 2.6 Sample size

The sample size was calculated using the single-proportion formula *n* = *Z*^2^(1−*α*/2)·*P*(1−*P*)/*d*^2^, with a two-sided significance level *α* of 0.05 (*Z* = 1.96). Based on the previous meta-analyses conducted for Chinese mothers' isolates (2), the projected value of *P* was 0.65, and *d* was 0.10. The estimated need for typeable isolates was around 90. Taking into account the expected 10%–15% loss in reviving, at least 105 banked isolates were required. Over the course of the study, 132 banked isolates were available. This calculation was powered for the overall erythromycin non-susceptibility estimate; no *a priori* power calculation was performed for lineage-stratified comparisons, which were pre-specified as exploratory.

### 2.7 Statistical analysis

The continuous variables are shown in terms of medians with interquartile range, whereas the categorical variables are in terms of frequency and percentage. The MIC50 and MIC90 were the minimal concentrations that inhibit 50% and 90% of isolates, respectively. The proportions are in terms of Wilson Score 95% confidence interval. The sequence type diversity was measured through Simpson's Index of diversity, *D* = 1 − Σ*n_i_*(*n_i_* − 1)/[*N*(*N* − 1)]. Stratified comparisons included serotype III versus non-III isolates for erythromycin non-susceptibility and iMLSB, CC17/ST17 versus non-CC17 isolates for erythromycin non-susceptibility, iMLSB and effective clindamycin availability, and CC23 versus non-CC23 isolates for erythromycin non-susceptibility. These comparisons were performed with the use of Fisher's exact test using Bonferroni corrections with values of *p* = 0.025 for the two serotype III comparisons, *α* = 0.0167 for the three CC17/ST17 comparisons, and *p* = 0.05 for the one CC23 comparison. Exact *P* values are reported to three significant figures, and stratified comparisons are interpreted as hypothesis-generating rather than confirmatory, so that a comparison meeting or lying at the corrected threshold is not treated as definitive. The markers for high-risk lineages and the generated prophylaxis markers have been shown in frequencies with 95% confidence intervals. Analyses were performed in SPSS Statistics 29.0 (IBM Corp., Armonk, NY, USA), the minimum spanning tree was constructed in PHYLOViZ 2.0 (18), and figures were prepared in GraphPad Prism 10.3 (GraphPad Software, La Jolla, CA, USA).

## 3 Results

### 3.1 Isolates and maternal-neonatal characteristics

**Figure 1:**
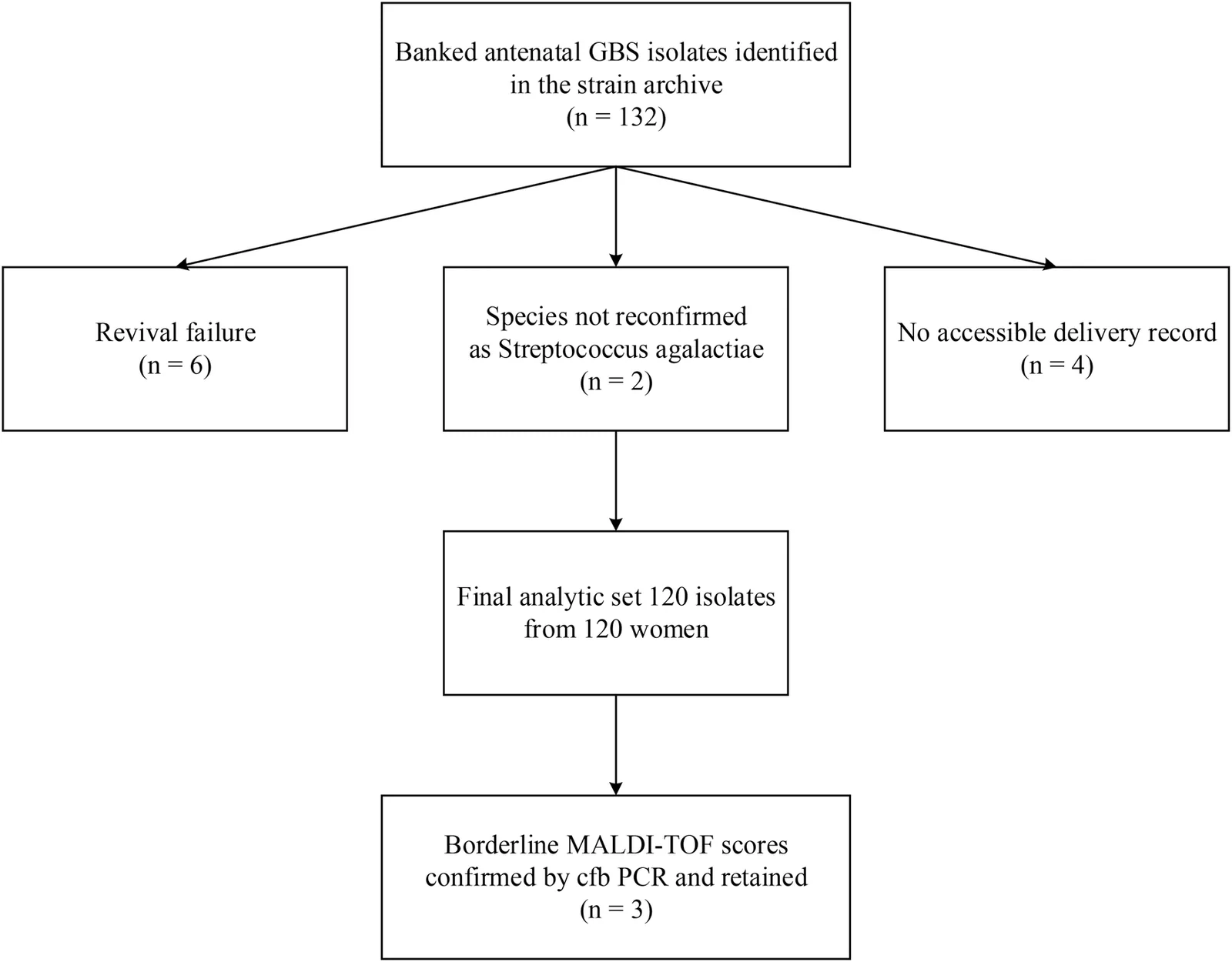
Flow of isolates from the strain archive to the analytic set.

Out of 132 isolates that were isolated and stored in the archives in the research period, 6 failed to grow when revived. A further 2 were excluded because the isolate could not be confirmed as *Streptococcus agalactiae*, and 4 could not be linked to a readable delivery record, leaving 120 isolates in the analysis group, each corresponding to a distinct woman (Figure 1). Three isolates with borderline MALDI-TOF scores were confirmed as *Streptococcus agalactiae* by *cfb* PCR and retained. Median maternal age was 29 years (IQR 27–32), median gravidity was 2 (IQR 1–3) and median parity was 1 (IQR 0–1). Antenatal testing was conducted at a median gestation age of 36.2 weeks (IQR 35.7–37.0). Vaginal deliveries formed 84 deliveries (70.6%) out of deliveries where mode of delivery was known, whereas Cesarean section deliveries were noted in 35 (29.4%) deliveries, with median gestation age at delivery of 39.4 weeks (IQR 38.3–40.0). Penicillin allergy was found in 14 patients (11.7%), among whom 6/12 (50.0%) patients where severity of penicillin allergy was known had high risk of anaphylaxis. Rupture-to-delivery interval had a median of 5.6 h (IQR 3.6–10.1); prolonged rupture of membranes occurred in 12 women (10.0%), intrapartum fever in 7 (5.8%), clinical chorioamnionitis in 4 (3.3%), preterm delivery in 4 (3.3%), neonatal intensive care unit admission in 9 (7.5%), and neonatal sepsis evaluation in 11 (9.2%). Complete demographic, obstetric, intrapartum prophylaxis and neonatal details are provided in Table 2.

**Table 2:** Demographic, obstetric, intrapartum prophylaxis and neonatal characteristics of the study population.

Characteristic	Value	Missing
Final analytic isolates/women	120	0
Age, years, median (IQR)	29 (27–32), *n* = 119	1
Gravidity, median (IQR)	2 (1–3), *n* = 119	1
Parity, median (IQR)	1 (0–1), *n* = 119	1
Screening week, median (IQR)	36.2 (35.7–37.0), *n* = 118	2
Delivery week, median (IQR)	39.4 (38.3–40.0), *n* = 119	1
Vaginal delivery	84 (70.6% of recorded)	1
Caesarean delivery	35 (29.4% of recorded)	1
Rupture-to-delivery interval, h, median (IQR)	5.6 (3.6–10.1)	0
Prolonged rupture of membranes ≥18 h	12 (10.0%)	0
Intrapartum fever	7 (5.8%)	0
Clinical chorioamnionitis	4 (3.3%)	0
Preterm delivery <37 weeks	4 (3.3%)	0
Penicillin allergy	14 (11.7%)	0
High-risk allergy	6 (50.0% of allergic women with recorded severity)	2
First-dose-to-delivery interval, h, median (IQR)	5.3 (3.3–8.6), *n* = 106	3
IAP first-dose interval >=4 h	69 (65.1% of prophylaxis-exposed women with recorded interval)	3
Neonatal intensive care unit admission	9 (7.5%)	0
Neonatal sepsis evaluation	11 (9.2%)	0

### 3.2 Serotype distribution and theoretical hexavalent serotype coverage

The 120 analytic isolates spanned nine capsular types plus two non-typeable isolates. Serotype III accounted for 35.8% (95% CI 27.8–44.7) of the collection and was the most common single type, followed by Ia (26.7%, 95% CI 19.6–35.2), Ib (16.7%, 95% CI 11.1–24.3) and V (6.7%, 95% CI 3.4–12.6). Types II and IV together accounted for nine of 120 isolates (7.5%; 95% CI 4.0–13.6), while the non-vaccine types VI to VIII together with the non-typeable strains accounted for eight of 120 (6.7%; 95% CI 3.4–12.6). The 112 isolates belonging to the six vaccine target types corresponded to a theoretical serotype coverage of 94.9% (95% CI 89.3–97.6) among the 118 typeable isolates. The complete distribution is shown in Table 3.

**Table 3:** Serotype distribution and hexavalent vaccine target status.

Serotype	Isolates	Proportion
III	43	35.8% (95% CI 27.8–44.7)
Ia	32	26.7% (95% CI 19.6–35.2)
Ib	20	16.7% (95% CI 11.1–24.3)
V	8	6.7% (95% CI 3.4–12.6)
II	5	4.2% (95% CI 1.8–9.4)
IV	4	3.3% (95% CI 1.3–8.3)
VI	3	2.5% (95% CI 0.9–7.1)
VIII	2	1.7% (95% CI 0.5–5.9)
VII	1	0.8% (95% CI 0.1–4.6)
NT	2	1.7% (95% CI 0.5–5.9)

### 3.3 Sequence types and clonal complexes

**Figure 2:**
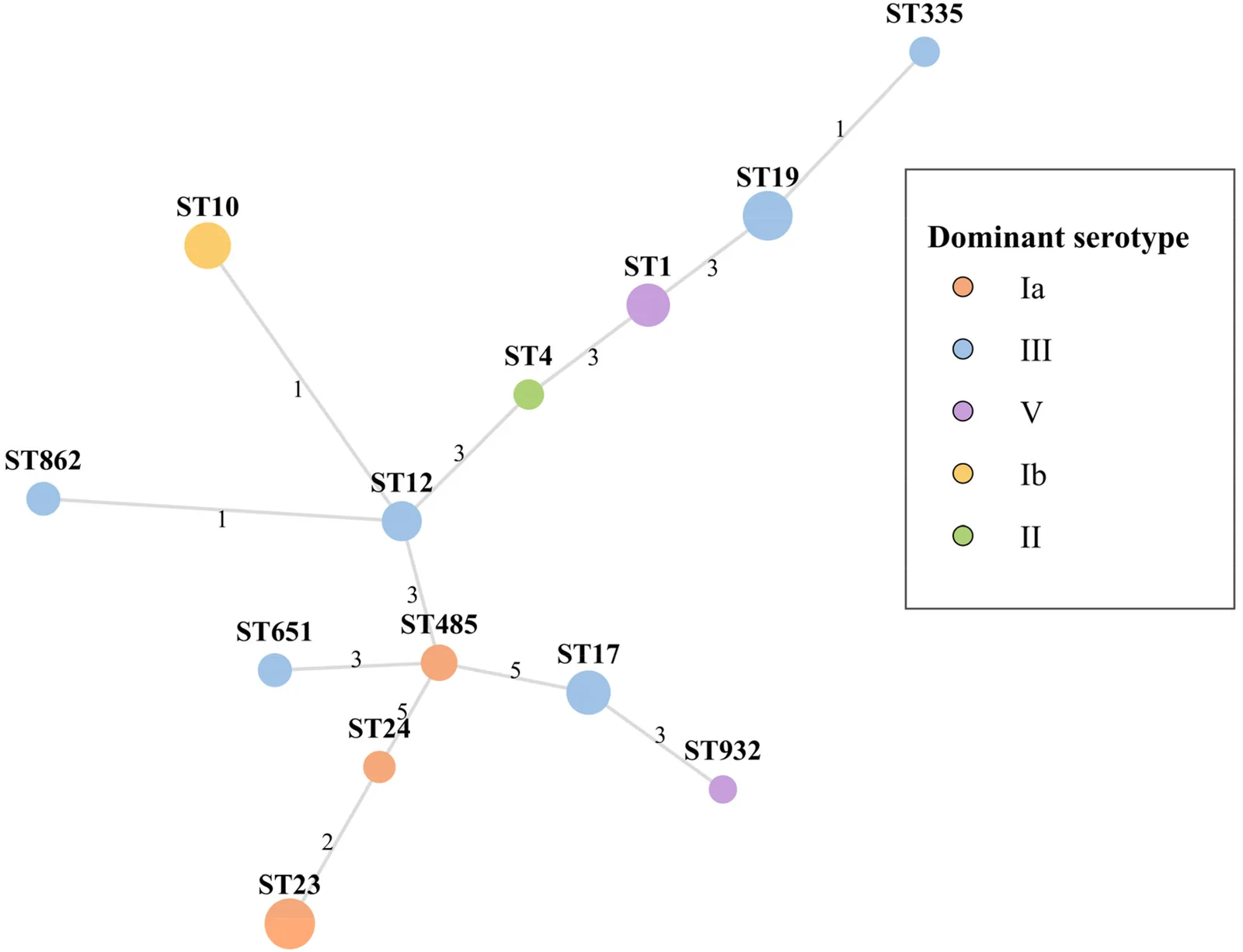
Minimum spanning tree of sequence types.

The MLST scheme divided the analysis sample into 13 sequence types without any discovery of novel sequence type. These sequence types were distributed across seven clonal complexes, of which CC19 (*n* = 28), CC23 (*n* = 26), CC10 (*n* = 26), CC1 (*n* = 18) and CC17 (*n* = 14) predominated, while CC485 (*n* = 6) and CC4 (*n* = 2) were infrequent. Simpson's index of diversity for sequence types across the collection was *D* = 0.880. ST17 accounted for 11.7% of all isolates (95% CI 7.1–18.6) and for 30.2% (95% CI 18.6–45.1) of isolates carrying capsular type III, while ST19 and ST23 accounted for 18.3% (95% CI 12.4–26.2) and 19.2% (95% CI 13.1–27.1) of the collection, respectively. Sequence types other than ST23, ST19, ST10, ST17 and ST1 were each represented by fewer than 10 isolates and are presented descriptively. In this isolate set, CC17 comprised ST17 only. The prevalence of different strains within the sequence types and their corresponding clonal complexes and capsule types is shown in Table 4. Within the minimum spanning tree, the dominant clusters were those of CC19, CC23, CC10, CC1, and CC17, wherein CC17 was denoted as ST17 and separated from CC19 and CC23 (Figure 2).

**Table 4:** Sequence types with associated clonal complex and capsular type.

ST	Clonal complex	Isolates	Serotype distribution
ST23	CC23	23	Ia:18, III:2, II:1, NT:1, Ib:1
ST19	CC19	22	III:18, Ia:2, V:1, VIII:1
ST10	CC10	17	Ib:13, V:1, II:1, Ia:1, NT:1
ST17	CC17	14	III:13, IV:1
ST1	CC1	13	V:5, Ia:4, II:2, Ib:1, VII:1
ST12	CC10	9	Ib:4, III:4, VIII:1
ST485	CC485	6	Ia:5, VI:1
ST651	CC19	4	III:3, IV:1
ST862	CC1	4	IV:2, III:2
ST24	CC23	3	Ia:2, VI:1
ST335	CC19	2	III:1, VI:1
ST4	CC4	2	Ib:1, II:1
ST932	CC1	1	V:1

### 3.4 Antimicrobial susceptibility and macrolide-lincosamide resistance phenotypes

All isolates were fully susceptible to penicillin (MIC50 0.06 µg/mL, MIC90 0.12 µg/mL, range 0.03–0.12), ampicillin (MIC50 0.06 µg/mL, MIC90 0.12 µg/mL, range 0.03–0.12) and vancomycin (MIC50 0.5 µg/mL, MIC90 1 µg/mL, range 0.25–1). Erythromycin non-susceptibility was observed in 63.3% (95% CI 54.4–71.4), with MIC50 1 µg/mL and MIC90 32 µg/mL, ranging from 0.06 to 64 µg/mL. Before D-test correction, the nominal clindamycin resistance rate was 43.3% (95% CI 34.8–52.3), with MIC50 0.25 µg/mL and MIC90 16 µg/mL, ranging from 0.03 to 32 µg/mL. Levofloxacin non-susceptibility was found in 27.5% (95% CI 20.3–36.1), comprising 10.0% intermediate and 17.5% resistant isolates (MIC50 1 µg/mL, MIC90 8 µg/mL, range 0.5–16), and tetracycline resistance in 71.7% (95% CI 63.0–79.0; MIC50 16 µg/mL, MIC90 64 µg/mL, range 0.5–64). Categorical proportions are shown in Table 5 and the complete MIC distributions in Table 6. MICs for *Streptococcus pneumoniae* ATCC 49619 remained within the CLSI quality-control ranges in all testing batches.

**Table 5:** MIC50, MIC90, MIC ranges and susceptibility categories.

Drug	MIC50	MIC90	MIC range	S	I	R
Penicillin	0.06	0.12	0.03–0.12	120	0	0
Ampicillin	0.06	0.12	0.03–0.12	120	0	0
Vancomycin	0.5	1	0.25–1	120	0	0
Erythromycin	1	32	0.06–64	44	5	71
Clindamycin	0.25	16	0.03–32	68	0	52
Levofloxacin	1	8	0.5–16	87	12	21
Tetracycline	16	64	0.5–64	34	0	86

MIC values in µg/mL. S/I/R categories assigned according to CLSI M100 breakpoints for β-haemolytic streptococci.

**Table 6:** Complete MIC distributions by two-fold dilution.

Drug	0.03	0.06	0.12	0.25	0.5	1	2	4	8	16	32	64
Penicillin	25	67	28									
Ampicillin	27	54	39									
Vancomycin				34	53	33						
Erythromycin		19	10	15	5	14	13	12	3	16	5	8
Clindamycin	14	13	22	19		8	10	12	8	7	7	
Levofloxacin					49	35	3	12	11	10		
Tetracycline					10	12	12		17	24	21	24

MIC values are shown in µg/mL. Columns represent two-fold dilutions; blank cells denote no isolate at that value.

**Figure 3:**
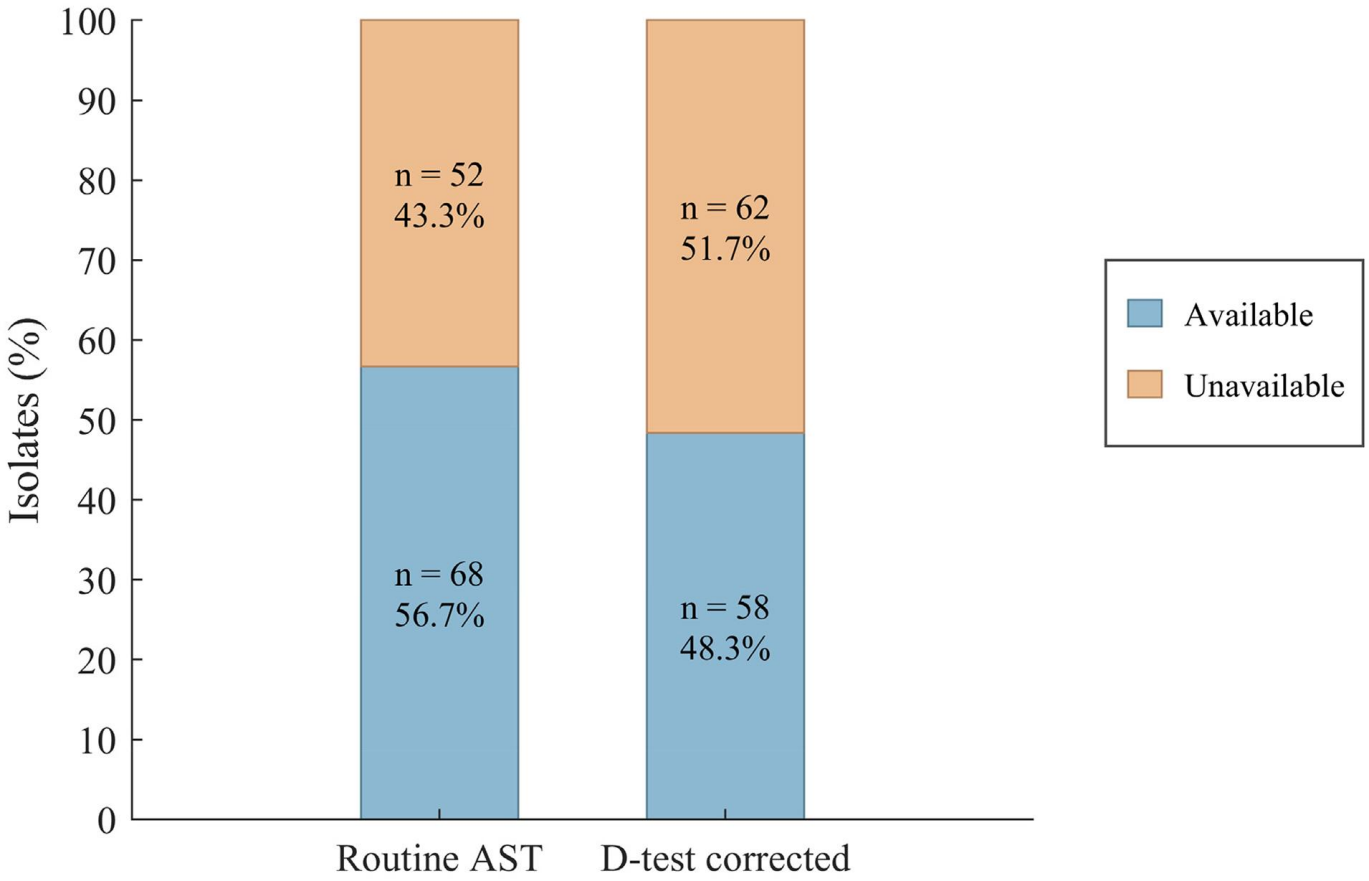
Effective clindamycin availability before and after D-test correction.

All erythromycin non-susceptible isolates (*n* = 76) were subjected to the D-test. The L phenotype (*n* = 4) was observed directly from the routine results among erythromycin-susceptible, clindamycin-resistant isolates. Across the analytic set, the cMLSB phenotype accounted for 40.0% (95% CI 31.7–48.9), the iMLSB phenotype for 8.3% (95% CI 4.6–14.7), the M phenotype for 15.0% (95% CI 9.7–22.5) and the L phenotype for 3.3% (95% CI 1.3–8.3). With susceptibility reporting alone, 56.7% (95% CI 47.7–65.2) of the analysis group would be considered to be clindamycin-susceptible. Reclassifying the 10 iMLSB isolates as resistant for the purpose of intrapartum prophylaxis reduced the effective availability of clindamycin to 48.3% (95% CI 39.6–57.2), a reduction of 8.3 percentage points (Figure 3).

### 3.5 Resistance genes and distribution of resistance by serotype and clonal complex

Among erythromycin non-susceptible isolates, *ermB* was the most frequently detected resistance gene, accounting for 56.6% (43/76; 95% CI 45.4–67.1), followed by *mefA/E* at 27.6% (21/76; 95% CI 18.8–38.6) and *ermA/ermTR* at 14.5% (11/76; 95% CI 8.3–24.1). The lincosamide resistance genes *lnuB* and *lsaC* were not detected among erythromycin non-susceptible isolates but were present in 2 and 1 L-phenotype isolates, respectively. *ermB* was most often found in cMLSB isolates, whereas *ermA/ermTR* was present in 5 of 10 iMLSB isolates (50.0%) as against 6 of 48 cMLSB isolates (12.5%), and *mefA/E* in 13 of 18 M-phenotype isolates (72.2%). Phenotype-genotype discrepancies were found in 26 isolates, falling into four categories: cMLSB or iMLSB isolates without a detectable *erm* gene (*n* = 11), M-phenotype isolates without *mefA/E* (*n* = 5), an L-phenotype isolate without *lnuB* or *lsaC* (*n* = 1), and erythromycin-susceptible isolates carrying a macrolide resistance determinant (*n* = 9); isolate-level detail for all 26 is given in Table 7. The complete phenotype-gene cross tabulation is given in Table 8; 11 isolates (9 cMLSB and 2 iMLSB) carried more than one screened gene. All PCR positive amplicons yielded sequences matching the predicted sequence on bidirectional sequencing.

**Table 7:** Isolate-level detail of the 26 phenotype-genotype discordant isolates.

ID	Serotype	ST	CC	Phenotype	D-test	ERY MIC	CLI MIC	Gene(s)	Category
GBS005	III	ST335	CC19	cMLSB	negative	16	16	none	i
GBS009	Ia	ST485	CC485	cMLSB	negative	16	2	none	i
GBS018	Ia	ST1	CC1	M	negative	64	0.12	none	ii
GBS023	Ib	ST10	CC10	L	not_done	0.25	16	none	iii
GBS025	III	ST651	CC19	ERY-S	not_done	0.25	0.12	*mefA/E*	iv
GBS026	III	ST17	CC17	M	negative	16	0.12	none	ii
GBS029	III	ST17	CC17	iMLSB	positive	1	0.03	*mefA/E*	i
GBS034	III	ST17	CC17	M	negative	8	0.25	*ermB*	ii
GBS036	V	ST1	CC1	cMLSB	negative	2	2	none	i
GBS041	NT	ST23	CC23	ERY-S	not_done	0.06	0.12	*mefA/E*	iv
GBS054	Ib	ST10	CC10	ERY-S	not_done	0.06	0.12	*mefA/E*	iv
GBS059	Ib	ST12	CC10	cMLSB	negative	2	4	none	i
GBS065	Ia	ST23	CC23	ERY-S	not_done	0.06	0.25	*mefA/E*	iv
GBS069	VI	ST24	CC23	cMLSB	negative	16	16	none	i
GBS072	III	ST862	CC1	ERY-S	not_done	0.06	0.25	*mefA/E*	iv
GBS073	Ib	ST10	CC10	iMLSB	positive	16	0.03	none	i
GBS078	Ia	ST10	CC10	ERY-S	not_done	0.25	0.03	*ermB*	iv
GBS081	Ia	ST23	CC23	ERY-S	not_done	0.25	0.06	*mefA/E*	iv
GBS087	III	ST17	CC17	iMLSB	positive	1	0.12	none	i
GBS091	Ia	ST23	CC23	ERY-S	not_done	0.06	0.25	*ermA/TR*	iv
GBS092	III	ST12	CC10	cMLSB	negative	1	4	none	i
GBS095	VI	ST485	CC485	M	negative	64	0.12	none	ii
GBS103	Ia	ST23	CC23	ERY-S	not_done	0.12	0.25	*ermA/TR*	iv
GBS105	Ia	ST1	CC1	cMLSB	negative	64	4	none	i
GBS108	Ia	ST485	CC485	M	negative	64	0.12	none	ii
GBS117	III	ST17	CC17	iMLSB	positive	4	0.06	none	i

Categories: (i) MLSB phenotype without a detectable *erm* gene; (ii) M phenotype without *mefA/E*; (iii) L phenotype without *lnuB/lsaC*; (iv) erythromycin-susceptible isolate carrying a macrolide resistance determinant. MIC values are shown in µg/mL.

**Table 8:** Resistance genes across macrolide-lincosamide resistance phenotypes.

Phenotype	*n*	*ermB*	*ermA/ermTR*	*mefA/E*	*lnuB*	*lsaC*
cMLSB	48	38	6	5	0	0
iMLSB	10	3	5	3	0	0
M	18	2	0	13	0	0
L	4	0	0	0	2	1
ERY-S	40	1	2	6	0	0

Determinant counts are not mutually exclusive; some isolates carried more than one screened gene.

Phenotypes resistant to erythromycin were separated by capsular type and clonal complex and compared against the rest of the group; for example, serotype III and CC17 were compared to the rest. Resistance to erythromycin was found in 60.5% (26/43) of serotype III strains and 64.9% (50/77) of the non-III strains (*P* = 0.694; Fisher's exact test), and the iMLSB phenotype was found in 7.0% (3/43) and 9.1% (7/77), respectively (Fisher's exact test *P* = 1.000). Neither comparison met the Bonferroni-adjusted threshold of 0.025. Erythromycin non-susceptibility was observed in 85.7% (95% CI 60.1–96.0) of CC17 isolates against 60.4% (95% CI 50.9–69.2) of non-CC17 isolates (Fisher's exact test *P* = 0.080), and the iMLSB phenotype in 28.6% (95% CI 11.7–54.6) against 5.7% (95% CI 2.6–11.8) (Fisher's exact test *P* = 0.0166). This was the only stratified comparison to reach the Bonferroni-adjusted threshold of 0.0167, and it did so only marginally. CC23 had a lower erythromycin non-susceptibility rate when compared to non-CC23 groups [34.6% [9/26] versus 71.3% [67/94]; Fisher's exact test *P* = 0.001]. Complete stratified proportions are shown in Table 9.

**Table 9:** Resistance phenotypes by serotype and clonal complex.

Stratum	Group	*n*	ERY non-S	cMLSB	iMLSB	M	L
Serotype	III	43	26	15	3	8	0
Serotype	Ia	32	15	6	3	6	3
Serotype	Ib	20	14	10	3	1	1
Serotype	V	8	7	6	0	1	0
Serotype	II	5	4	4	0	0	0
Serotype	IV	4	4	2	1	1	0
Serotype	VI	3	3	2	0	1	0
Serotype	VIII	2	2	2	0	0	0
Serotype	VII	1	0	0	0	0	0
Serotype	NT	2	1	1	0	0	0
Clonal complex	CC19	28	17	12	0	5	0
Clonal complex	CC23	26	9	3	2	4	3
Clonal complex	CC10	26	17	12	3	2	1
Clonal complex	CC1	18	14	12	1	1	0
Clonal complex	CC17	14	12	5	4	3	0
Clonal complex	CC485	6	5	2	0	3	0
Clonal complex	CC4	2	2	2	0	0	0

ERY non-S, erythromycin non-susceptible.

### 3.6 High-risk lineage and clindamycin availability

The EOD-associated CC17/ST17 lineage accounted for 14 of 120 isolates (11.7%; 95% CI 7.1–18.6) and for 14 of 118 typeable isolates (11.9%; 95% CI 7.2–18.9). Among CC17/ST17 isolates, 12 were erythromycin non-susceptible, comprising 5 cMLSB, 4 iMLSB and 3 M-phenotype isolates. The remaining 2 were erythromycin-susceptible and clindamycin-susceptible. The iMLSB phenotype accounted for 28.6% (4/14) of CC17/ST17 isolates, compared with 8.3% (10/120) of the full analytic set and 5.7% (6/106) of non-CC17 isolates. All three iMLSB isolates of capsular type III belonged to CC17, and no serotype III isolate outside CC17 showed the iMLSB phenotype, so that within serotype III the inducible signal tracked with the clonal background rather than the capsule. Effective clindamycin availability was 35.7% (5/14; 95% CI 16.3–61.2) in CC17/ST17 isolates and 50.0% (53/106; 95% CI 40.6–59.4) in non-CC17 isolates (Fisher's exact test *P* = 0.399), which did not meet the Bonferroni-adjusted threshold of 0.0167. The corresponding confidence intervals were wide.

### 3.7 Intrapartum prophylaxis actually administered and neonatal outcomes

The intrapartum prophylaxis regimen documented from the delivery record included penicillin, ampicillin, or cefazolin in 98 women (81.7%), clindamycin in 8 women (6.7%), vancomycin in 3 women (2.5%), and no prophylaxis in 11 women (9.2%). Of the 14 women with reported penicillin allergy, 6 were documented as high risk for anaphylaxis. Clindamycin was given to 8 of these women, vancomycin to 3 and cefazolin to 3; of the 8 clindamycin exposures, 3 were in women whose documented allergy was not high risk, and 1 of these 3 isolates carried the L phenotype and was clindamycin-resistant. Of the 106 women exposed to prophylaxis for whom a first-dose interval was documented, the median interval from first dose to delivery was 5.3 h (IQR 3.3–8.6), and 69 (65.1%; 95% CI 55.6–73.5) had received the first dose at least 4 h before birth. Table 10 shows the entire cross-tabulation for the regime used in comparison to the D-test-defined phenotype of the isolate.

**Table 10:** Intrapartum regimen actually administered by D-test-defined phenotype of the colonizing isolate.

IAP regimen	*n*	cMLSB	iMLSB	M	L	ERY-S
penicillin	52	20	2	8	1	21
ampicillin	43	18	6	6	2	11
cefazolin	3	0	0	2	0	1
clindamycin	8	2	1	2	1	2
vancomycin	3	2	0	0	0	1
none	11	6	1	0	0	4

Effective clindamycin availability was examined in the three pre-specified denominators. Across all 120 isolates it was 48.3% (58/120; 95% CI 39.6–57.2). Among the 14 penicillin-allergic women it was 57.1% (8/14; 95% CI 32.6–78.6), and among the 6 high-risk-allergic women it was 50.0% (3/6). Among the 8 women who actually received clindamycin, 4 (50.0%; 95% CI 21.5–78.5) were colonised by an isolate confirmed by the D-test as iMLSB or phenotypically cMLSB or L, so that the administered agent was microbiologically inactive against the woman's own isolate; the remaining exposures involved M-phenotype isolates (*n* = 2) or erythromycin-susceptible, clindamycin-susceptible isolates (*n* = 2).

Clinical complication events were infrequent. Overall, prolonged rupture of membranes ≥18 h, intrapartum fever, clinical chorioamnionitis, neonatal intensive care unit admission and neonatal sepsis evaluation were recorded in 12, 7, 4, 9 and 11 mother-infant pairs, respectively. Among CC17/ST17 isolates, one mother-infant pair each had prolonged rupture of membranes ≥18 h, intrapartum fever, clinical chorioamnionitis, neonatal intensive care unit admission and neonatal sepsis evaluation. These are unadjusted descriptive counts; no statistical testing was performed, and no association should be inferred.

One newborn in the analytical group developed early onset GBS infection during the first 7 days of its life. The mother had been colonized with a serotype Ia/ST23 strain of the pathogen with iMLSB phenotype, and she had taken clindamycin for prophylactic purposes 3.6 h prior to the birth. The neonate's blood sample proved to be positive for GBS. Cerebrospinal fluid culture was not obtained. The neonatal isolate was not available for comparative typing.

## 4 Discussion

Susceptibility surveillance overestimated the proportion of clindamycin available for use as prophylaxis during delivery by 8.3 percentage points in this cohort. This difference has direct implications for the choice of intrapartum prophylaxis, because among women treated with clindamycin during delivery, half were colonized with isolates against which the drug lacked *in-vitro* activity. Inducible resistance in maternal colonizing bacteria had been previously noted using the D-test at similar frequencies (13, 22), and resistance estimates to clindamycin have increased over successive time periods (11). Because many estimates rely on routine susceptibility testing, they can underestimate the proportion of isolates unsuitable for clindamycin prophylaxis, especially where macrolide resistance is common. Underestimation is therefore inherent to clindamycin reporting rather than specific to one location. L-phenotype isolates raise the same practical issue from the opposite direction: they are detected by routine testing, but should still be counted as clindamycin-unavailable for prophylaxis.

The capsular composition observed here replicates the general trend found in Chinese maternal isolates where serotype III is most common followed by serotypes Ia and Ib (2, 13). The proportion of serotype III and the presence of types VI to VIII are also consistent with geographical variation in capsular distribution over time (23, 24). The theoretical hexavalent serotype coverage should be read as a ceiling rather than expected vaccine effectiveness. Its interpretation is constrained because colonizing and invasive strain populations differ; neonatal infection isolates contain more ST17/CC17 than contemporaneous maternal colonizing isolates (25). Serotype coverage from colonizing strains therefore does not directly estimate disease prevention, and capsular matching does not by itself establish protection, which also depends on immunogenicity, antibody transfer and serotype-specific efficacy (9). Non-vaccine and non-typeable isolates also keep the ceiling below 100% and could become more visible under vaccine selection pressure. Maternal immunization has an advantage over antibiotics in that it is independent of resistance and sensitivity issues, and conjugate and protein vaccines are being evaluated in clinical studies (26, 27).

The proportion of CC17/ST17 in this colonizing collection was greater than that in previous studies of Chinese maternal isolates (2) yet substantially lower than what it is among invasive neonatal isolates (7, 25). This is why lineage data from colonizing isolates should not be interpreted as direct evidence of neonatal risk. The present data instead show that inducible resistance was enriched in the EOD-associated CC17/ST17 lineage. This was the only stratified comparison to marginally meet the adjusted threshold. Therefore, a lineage more often associated with invasiveness was also more likely to be falsely interpreted as clindamycin-susceptible. A biological link is plausible because integrative and conjugative elements replacing pilus island 1 in serotype III/CC17 strains can carry both *erm* and *tet* determinants together (8, 22, 28). This result should be interpreted cautiously. CC17/ST17 included only 14 isolates, the confidence intervals were wide, and serotype III itself did not show increased erythromycin non-susceptibility or iMLSB frequency. The signal therefore lies at the clonal-complex level rather than the capsular-type level, and serotype should not be used as its proxy. Clinical complication events were few and did not establish an association between resistance phenotype and maternal or neonatal outcome. In addition, *hvgA* was not genotyped and CC17 membership was inferred by MLST alone. These data do not justify changing screening practice, but they support further lineage-aware surveillance.

The discordance between resistance phenotype and genotype in 26 isolates warrants comment, because it bears on how the present genotypic data should be read. The largest category comprised erythromycin-susceptible isolates that nonetheless carried a macrolide determinant, most often *mefA/E*; a low level of efflux-pump expression can leave the erythromycin MIC within the susceptible range, so that the gene is present but not phenotypically apparent. A second category comprised cMLSB or iMLSB isolates in which no *erm* gene was amplified; because the panel targeted *ermB* and the *ermA/ermTR* subclass only, resistance mediated by other methylases or by 23S rRNA and ribosomal-protein mutations would not have been detected. The single L-phenotype isolate without *lnuB* or *lsaC* points in the same direction, as lincosamide inactivation can be conferred by determinants outside the panel. These explanations are not mutually exclusive and cannot be distinguished without sequencing, which is why the discordant isolates are reported descriptively rather than as evidence of any single mechanism.

D-testing all maternal isolates non-susceptible to erythromycin is a direct and practical approach, requiring only an additional disc arrangement from an already isolated specimen. Without it, a subset of isolates may be reported as clindamycin-susceptible despite inducible resistance. The result should be interpreted, not merely reported, because a clindamycin MIC read as susceptible is misleading when the D-test is positive, and obstetricians need to know whether the antibiotic is suitable for prophylaxis. The penicillin-allergy pathway also requires careful documentation, because cefazolin is indicated for women whose reported reaction is not associated with a high risk of anaphylaxis or whose reaction severity is unknown (6). It is even indicated for women reporting IgE-mediated reactions (29). Clindamycin and vancomycin are often used inappropriately in women with low-risk allergy (30, 31). Restricting vancomycin to those who truly have a high risk of allergy and their isolate is MLSB resistant would narrow vancomycin use and improve stewardship. The D-test helps to avoid ineffective clindamycin exposure and unnecessary vancomycin exposure. Local iMLSB prevalence can also help estimate obstetric vancomycin needs more accurately than allergy prevalence alone.

Several limitations constrain the interpretation of the conclusions reached. This retrospective study was performed at one centre using a strain bank, and local resistance patterns, referral practice and laboratory workflow may limit generalizability to other regions or health care systems (32). Those strains that could not be revived or species confirmed remain of unknown serotype and resistance profile, and thus cannot be considered randomly lost. The variables recorded for the first-dose interval and allergy severity were incomplete, and because these parameters determine the denominator used to estimate retrospective microbiological ineffectiveness, the estimates could be either underestimated or overestimated. The single early-onset disease case allows only description; it cannot establish a causal relation between inducible resistance and neonatal disease. Whole-genome sequencing was not performed, and the PCR panel covered only selected resistance determinants rather than all reported mechanisms, which limits interpretation of phenotype-genotype discordance. Virulence determinants such as *hvgA* were not genotyped, and colonization rate and colonization load were not study outcomes. Future work should include prospective multicentre cohorts linking microbiologically ineffective prophylaxis to neonatal colonization and early-onset disease, together with whole-genome sequencing of discordant and CC17/ST17 isolates.

## 5 Conclusions

Maternal colonizing GBS in this centre remained fully susceptible to penicillin, ampicillin, and vancomycin, while erythromycin non-susceptibility was common. Serotypes III, Ia and Ib predominated, and the theoretical hexavalent serotype coverage was high but constrained by non-vaccine and non-typeable isolates. The EOD-associated CC17/ST17 lineage was enriched for the inducible clindamycin-resistance phenotype. Routine susceptibility reporting overestimated effective *in-vitro* clindamycin availability, and clindamycin prophylaxis already administered at this site was microbiologically inactive against the colonizing isolate in a subset of cases. D-testing of every erythromycin non-susceptible maternal isolate, together with reporting that states whether clindamycin is suitable for intrapartum use, would better align microbiological results with prophylaxis decisions in penicillin-allergic women. Continued serotyping and sequence typing of colonizing isolates would also provide a local baseline for future vaccine introduction.

## Data Availability

The original contributions presented in the study are included in the article/Supplementary Material, further inquiries can be directed to the corresponding author.
